# Outcome After Anterior Cruciate Ligament Revision

**DOI:** 10.1007/s12178-019-09571-5

**Published:** 2019-07-08

**Authors:** Alexandra Horvath, Eric Hamrin Senorski, Olof Westin, Jón Karlsson, Kristian Samuelsson, Eleonor Svantesson

**Affiliations:** 1 Department of Orthopaedics, Sahlgrenska University Hospital, Mölndal, Sweden; 2 Department of Orthopaedics, Institute of Clinical Sciences, Sahlgrenska Academy, University of Gothenburg, PO Göteborgsvägen 31, SE-431 80 Mölndal, Gothenburg Sweden; 3 Department of Health and Rehabilitation, Institute of Neuroscience and Physiology, Sahlgrenska Academy, University of Gothenburg, Gothenburg, Sweden

**Keywords:** Anterior cruciate ligament, Revision, Concomitant injury, Graft choice, Patient-reported outcome, Return to sport

## Abstract

**Purpose of Review:**

To describe the current literature related to anterior cruciate ligament (ACL) revision in terms of surgical aspects, graft choices, concomitant injuries, patient-reported outcome, return to sport, and objective measurement outcome.

**Recent Findings:**

An ACL rupture is a common knee injury, and the number of primary ACL reconstructions is increasing, implying a subsequent increase of ACL revisions in the future. It is widely accepted that an ACL revision is surgically challenging with a myriad of graft options to choose from. In many cases, simultaneous injuries to the index limb including meniscal and chondral lesions, respectively, are observed in the setting of a secondary ACL injury. Furthermore, the general understanding is that an ACL revision results in inferior outcome compared with a primary ACL reconstruction.

**Summary:**

Surgical treatment of an ACL revision can be performed as one-stage or two-stage procedure depending on, for example, the presence of limb malalignments, concomitant injuries, and tunnel widening. Nonirradiated allografts and autologous patella tendon, hamstring tendon, and quadriceps tendon are feasible options for ACL revision. Concomitant injuries to the affected knee such as intraarticular chondral lesions are more common in the setting of an ACL revision compared with primary ACL reconstruction while a lower presence of concomitant meniscal pathology is reported at ACL revision. Patients undergoing ACL revision have lower clinical and patient-reported outcome and lower rates of return to sport when compared with primary ACL surgery cases. However, long-term follow-ups with large study cohorts evaluating outcome of ACL revision are limited. Further research is needed to confirm the present findings of this review.

## Introduction

Injury to the anterior cruciate ligament (ACL) is common, with an estimated incidence of 200,000 primary ACL ruptures annually only in the USA [1, 2]. The number of patients undergoing primary ACL reconstruction is increasing [3, 4], and it is likely that ACL revision will increase in the future [3, 5]. Previous studies have demonstrated that the most common causes leading to an ACL revision are technical errors, traumatic reinjury, and biological factors [5–7, 8•]. The general understanding is that an ACL revision results in inferior clinical and patient-reported outcome compared with a primary ACL reconstruction [9, 10•, 11•]. It is also widely accepted that an ACL revision poses surgical and biological challenges for the orthopedic surgeon. In-depth knowledge of the surgical technique and factors that affect outcome is pivotal in order to optimize outcome and to set realistic expectations for the future knee function in patients who opts for ACL revision. This clinical review is aimed at summarizing the current evidence related to ACL revision in terms of surgical principles, graft choice, concomitant injuries, clinical and patient-reported outcome, and return to sport (RTS).

## Material and Methods

In December 2018, we searched PubMed for papers on one- or two-stage ACL revision, graft choice, concomitant injuries, and outcome related to ACL revision. The studies had to include individuals who were 13 years or older and had undergone ACL revision. To identify outcome studies on ACL revision, we used the keywords *anterior cruciate ligament* and *revision* and *outcome*. The search strategy resulted in 403 studies in total, for which all abstracts were reviewed for relevance. Of the reviewed abstracts, 49 abstracts were determined as relevant for the study purpose, and these studies were reviewed in full text. The studies were stratified according to the main study topic as follows: 5 studies were on one- or two-stage ACL revision, 4 studies on graft choice, 10 studies on concomitant injuries, and 30 studies on different outcome variables. Additional literature was identified via hand-searching of the reference lists of relevant studies. Furthermore, reviews, meta-analysis, or clinical commentaries were also reviewed to identify studies not found through the primary literature search.

This clinical review synthesized data across the a priori set topics “one- or two-stage ACL revision,” “graft choice for ACL revision,” “concomitant injuries in ACL revision,” and “outcome after ACL revision,” with additional subheadings to each topic created as considered necessary. Primarily, empirical data from studies comparing patients who had undergone primary reconstruction and revision ACL were extracted. Secondarily, empirical data from original studies and review papers summarizing evidence on outcomes were extracted.

## Results

### One- and Two-Stage Anterior Cruciate Ligament Revision

The indications for one-stage ACL revision can be applied when the original bone tunnels are far away from the native footprints with no presence of tunnel enlargement or concomitant lesions to the affected limb including varus or valgus malalignment, cartilage deficiency, or meniscal injury [12]. A two-stage procedure can be performed in the presence of arthrofibrosis, loss of range of motion (loss of flexion or extension more than 20° or 5°, respectively), bone tunnel interference, or bone tunnel enlargement (more than 14–16 mm) [12, 13]. A two-stage procedure is applied in approximately 6–9% of all ACL revisions [13, 14]. In the first phase of a two-stage revision, evaluation of the knee joint and concomitant injuries including meniscal and chondral lesions during the routine arthroscopy is performed. The primary ACL graft and its residual soft-tissue constituents are debrided, and previous hardware are only extracted if they interfere with the placement of the new bone tunnels [13]. Existing tunnels are then filled with synthetized, allogenous, or autogenous bone grafts derived from the tibia or iliac crest [12]. A computed tomography (CT) exam of the injured knee should be performed prior to the second phase in order to evaluate proper healing of the bone graft for future secure tunnel fixation. The second phase is performed 4–6 months after the first phase depending on the choice of bone graft, as incorporation of allogenous bone grafts is reported to have longer duration of healing compared with autogenous grafts [15]. In the second phase, an autograft or allograft ligament is utilized for the reconstruction of the ACL, and concomitant injuries such as meniscal or chondral defects are addressed. In specific, meniscal suturing and treatment of chondral lesions demonstrate superior healing in the presence of a concomitant ACL reconstruction since restoration of stability in the ACL injured knee decreases loading on the meniscus and cartilage and subsequently enable proper healing [16–18]. However, in cases with a reruptured ACL and concomitant meniscal injury where ACL revision and meniscectomy is planned, meniscal excision is performed when considered appropriate.

During a one-stage procedure, the existing graft is removed, and soft-tissue remnants of the graft are debrided. Previous anatomically positioned bone tunnels can often easily be revised. However, in the setting of tibial tunnels either too posteriorly or anteriorly placed compared with a femoral tunnel, the “divergent tunnel” concept can be applied. Specifically, a divergence of new and previous tunnels is created with the aid of redirecting away from the old tunnels, and the new tunnels are created in an anatomical fashion. As a consequence, the risk of tunnel convergence and “figure-eight” defects is minimized, and previous fixation devices may be left in situ as it otherwise is challenging to remove. Previously enlarged tunnels may be filled with bone grafts with the addition of interference screws on the opposite side of the bone graft [19]. In the presence of bony defects that cannot be addressed or if the creation of new tunnels is not possible, the one-stage technique may be converted to a two-stage procedure, as necessary [12].

### Graft Choice for Anterior Cruciate Ligament Revision

The decision-making for graft choice in ACL revision is complicated due to factors related to the primary ACL reconstruction, such as prior graft choice, the reason for ACL revision, and previous donor site morbidity. Data from one of the largest ACL revision cohorts at date, including 1205 ACL revision patients, demonstrate that patients who received an autologous graft for ACL revision were 2.78 times more likely to sustain a graft rupture compared with those who received an autograft [20•]. Published literature demonstrates that nonirradiated allografts for ACL revision result in similar outcomes compared with autografts, while a comparison irrespective of whether the allografts were nonirradiated or irradiated demonstrated that the use of allograft was associated with an increased anteroposterior laxity, more complications, and a higher reoperation rate compared with autografts [21]. High-quality studies on this topic are however few, with most studies being retrospective on heterogenous populations and outcome measurements [12, 21]. Conclusion making is also aggravated by studies including various types of allografts, different sterilization techniques, and unknown or variable age of the allograft donors which may impact the allograft quality. A recent review aimed to minimize such differences by performing a separate analysis limited to level II studies or higher and found a low and comparable failure rate between autogenous and allogenous grafts for ACL revision (4.1% compared with 3.6%, respectively) [22•].

With regard to autologous grafts for ACL revision, the use of patella tendon (PT) autograft has demonstrated suboptimal results which may possibly be explained by increased harvest site morbidity, resulting in more anterior knee pain and patellofemoral symptoms [23, 24]. However, the bone block at both ends of the PT autograft could be advantageous in settings where bone packing of tunnel widening is warranted or when attempting an off-set placement within previous bone tunnels. Nonetheless, an ipsilateral reharvest of the PT for ACL revision has been associated with inferior short-and long-term patient-reported outcome (PRO) [25, 26]. Concerns regarding reharvesting of the PT have also been raised since studies have reported an increased thickness and an incomplete healing of the remaining PT following harvest [25, 27, 28]. Such findings have, however, been contradicted by other studies showing full reconstitution of the donor site after PT harvest [29, 30], as well as successful clinical outcomes with PT reharvest for ACL revision [31, 32]. A reharvested PT can undergo remodeling and obtain ligamentous ACL characteristics with neovascularization and organization of the collagen when used for ACL revision, with successful restoration of knee joint stability [26]. The literature regarding reharvesting of the PT is limited by small study cohorts, and it seems evident that a complete reconstitution of the PT after being harvested for the primary ACL reconstruction cannot be assured. A PT reharvest may therefore result in a weakened ACL graft, as well as severe donor site morbidity. Thus, other graft choices, including a contralateral primary PT harvest, should be considered before aiming for reharvest of the PT in the setting of an ACL revision.

A contralateral autologous graft harvest for ACL revision could be advantageous in order to avoid further donor site morbidity in the injured limb. Studies on contralateral hamstring (HT) harvest for ACL revision report this as a feasible option [33], with a similar clinical and patient-reported outcome compared with both ipsilateral HT harvest and allograft [34–36].

In recent years, the quadriceps tendon (QT) has gained increasing attention for both primary reconstruction and ACL revision. The QT autograft can be reliably harvested, with and without bone block, with a robust tissue volume, low rate of donor site morbidity, and equivalent outcomes to other autograft types [37–39]. While the number of clinical studies investigating the use of QT autograft in ACL revision is few, a recent study evaluated the use of an ipsilateral QT autograft compared with contralateral HT autograft for ACL revision and found no differences in postoperative knee joint stability or PRO between the two [40].

In conclusion, nonirradiated allografts are valid options with high potential of a satisfactory outcome and are suitable in cases with complex knee laxity that need multiple-ligament reconstruction or when aiming to avoid further donor site morbidity. The use of PT autograft may result in slightly inferior patient-reported knee function compared with HT autograft, and a reharvest of the PT should only be performed after thorough consideration of other possible graft types and after assessing the degree of PT reconstitution. While a contralateral HT autograft is a valid option, caution should be taken in young females due to the increased risk of a contralateral ACL rupture observed in primary ACL reconstruction [41]. Successful outcomes with the use of QT autograft for primary ACL reconstruction suggest that it is an appropriate graft choice also for an ACL revision; however, future studies specifically investigating the use of QT in ACL revision cases are warranted.

### Concomitant Injuries in Anterior Cruciate Ligament Revision

Data from knee ligament registries worldwide demonstrate a high prevalence of concomitant intraarticular knee injuries in patients undergoing ACL revision. Specifically, concomitant chondral pathology is more common in ACL revision cases compared with a primary ACL reconstruction [42–46]. According to the literature, approximately 40–45% of the patients will have at least one concomitant cartilage injury at ACL revision [42, 43, 45, 47], which can be compared with 25–30% at primary ACL reconstruction [42, 43, 45]. One study from the Kaiser Permanente Anterior Cruciate Ligament Reconstruction Registry (KPACLRR) followed the same patient cohort from primary reconstruction to ACL revision and found that the prevalence of cartilage injury increased from 14.9 to 31.8% between the two events [46]. Progression of cartilage injuries from primary reconstruction to ACL revision has been strongly associated with meniscal resection at the primary ACL reconstruction [48]. Resection of more than one third of the lateral meniscus at primary ACL reconstruction increased the odds nearly 17-fold of progression of lateral compartment cartilage injury to ACL revision. In addition, even less than one third resection of the medial meniscus increased the odds 5-fold for progression in the medial compartment [48]. Hence, these findings support the importance of intact menisci for load distribution and suggest that meniscal resection at primary ACL reconstruction might cause irreversible damage to the underlying cartilage due to increase loads. However, the odds for the presence of a cartilage injury are increased in ACL revision compared with primary ACL reconstruction independently of meniscal status [49].

With regard to concomitant meniscal injuries, patients undergoing ACL revision are observed to have fewer meniscal injuries compared with primary ACL reconstruction cases [42, 43, 45, 46]. A study from the Swedish National Knee Ligament Registry reported fewer meniscal injuries at ACL revision compared with primary ACL reconstruction (33% compared with 42%) [43]. This is supported by data from the KPACLRR, where meniscal injuries were present in 53.2% at ACL revision compared with 60.8% at primary ACL reconstruction [45]. Interestingly, several studies have reported that lower prevalence of meniscal injuries at ACL revision is mainly a result of a decrease in lateral meniscal injuries, while the prevalence of medial meniscal injuries is similar between primary and revision ACL reconstruction [46, 49]. Thus, medial meniscus injuries are more common than lateral meniscus injuries in ACL revision [42, 44, 50, 51].

Concomitant injuries to a reruptured ACL have been associated with inferior outcome after ACL revision. In patients undergoing ACL revision, previous lateral meniscus resection and a grade 3 to 4 trochlear cartilage injury have been shown to have the most detrimental effects on outcome, with significantly increased odds for inferior outcome in the International Knee Documentation Committee (IKDC), Knee Injury and Osteoarthritis Outcome Score (KOOS), and Western Ontario and McMaster Universities Osteoarthritis Index (WOMAC) score [51]. In a midterm perspective (mean follow-up 4.6 years from ACL revision), grade 3 to 4 chondral pathology resulted in inferior patient-reported knee function, decreased activity level, and a lower RTS rate [50]. Lateral meniscal injury did not influence outcome; however, medial meniscal injury resulted in lower Marx activity level, KOOS-quality of life (QoL), and Single Assessment Numeric Evaluation (SANE) scores compared with patients without medial meniscal injury [50].

### Outcome After Anterior Cruciate Ligament Revision

#### Patient-Reported Outcome

Data from meta-analyses of the literature evaluating PRO following primary reconstruction and ACL revision demonstrate inferior PRO in patients undergoing an ACL revision [10•, 52]. Specifically, a recent systematic review of the Scandinavian knee ligament registers reported that ACL revision patients had inferior KOOS and European Quality of Life-5 dimensions (EQ-5D) compared with primary ACL reconstruction. In addition, similar improvement was demonstrated in patients with ACL revision in terms of KOOS subscales, 1 and 2 years after revision compared with patients undergoing primary reconstruction. However, it was more common for patients who underwent ACL revision to report lower KOOS-QoL score compared with the primary ACL reconstruction group [53•]. In a study on 552 athletes (primary (*n* = 479) versus revision (*n* = 55) ACL cases), the primary reconstruction group at 1-year follow-up had superior PRO compared with ACL revision patients in terms of IKDC and KOOS symptoms subscale. Additionally, function in activities of daily living (ADL) was significantly higher for patients undergoing primary ACL reconstruction compared with the ACL revision group, while no difference was observed in the Lysholm score [54]. One study by the Multicenter Orthopaedic Outcomes Network (MOON) cohort [9] evaluated 393 patients during a 2-year period. There were 364 cases with primary ACL reconstruction and 29 cases with an ACL revision. At 2 years, Marx activity level and IKDC were significantly different between the two groups with superior results in the primary ACL reconstruction group compared with the ACL revision group. Moreover, KOOS pain, sports/recreation, and QoL subscales were significantly lower in cases with ACL revision compared with primary ACL reconstruction [9]. With regard to surgical predictors of PRO following ACL revision, data from the Multicenter ACL Revision Study (MARS) group [11•] on 1205 patients (follow-up at 2 years) showed that patients without a notchplasty at the time of revision had inferior outcome with regard to IKDC, KOOS-ADL and QoL subscales, WOMAC stiffness subscale, and ADL scores compared with baseline values. In addition, a new tibial tunnel at the time of revision was associated with inferior KOOS-ADL and WOMAC-ADL compared with using the previous bone tunnel [11•]. In a cohort study on 109 patients undergoing ACL revision (average follow-up 4.9 years) of which 105 patients had undergone a one-staged revision and four a two-staged revision, patients returning to preinjury level of activity had higher Marx activity level, IKDC, and KOOS-QoL compared with those who did not [55]. Data from a study on 107 ACL revision patients (mean follow-up 72.9 ± 20.6 months) demonstrate improved Lysholm score and Tegner activity level compared with prior to ACL revision. In terms of IKDC, 65.4% assessed their knee as normal or nearly normal, while 34.6% considered their knee abnormal or severely abnormal [56].

Pooling of the current literature indicates improved PRO following ACL revision compared with what patients report preoperatively [36, 40, 57, 58], though the results appear inferior compared with primary ACL reconstruction [53•]. However, most studies evaluating outcome following ACL surgery compare primary ACL and revision ACL reconstruction, the latter one with small study samples which should be acknowledged when interpreting study results. Furthermore, cohorts of ACL revision patients also have small study populations, have short follow-up time, and are mostly retrospective. Future large cohort studies are warranted to establish the long-term outcome in patients who undergo ACL revision.

#### Return to Sport

**Fig. 1:**
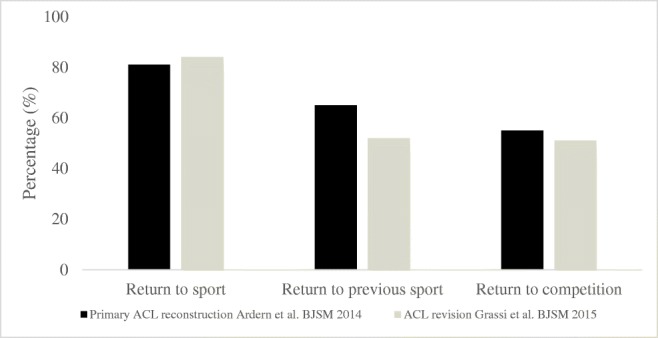
The proportion of patients returning to sport after primary and revision ACL reconstruction as reported by Ardern et al. [67] and Grassi et al. [68]. ACL—anterior cruciate ligament, BJSM—*British Journal of Sports Medicine*

The overall RTS rate appears lower after ACL revision compared with primary ACL reconstruction (Fig. 1) [10•, 59, 60]. The return to any sport and return to competition are similar for patients undergoing primary and revision ACL reconstruction, suggesting a good prognosis for patients to maintain an active lifestyle. However, the rate of patients returning to preinjury sport is higher for patients undergoing primary reconstruction compared with ACL revision [10•, 54, 59, 60] (Fig. 1), meaning that patients may need to adapt their level of sport since the return to preinjury sport rate is lower after ACL revision compared with primary ACL reconstruction. The outcome of return to sport is many times used as a reflection of treatment success; however, one must acknowledge that the additional trauma caused during graft rupture and ACL revision may result in such intraarticular injuries that participation in sport is not possible. Patients may also actively choose or be recommended by their orthopedic surgeon or physical therapist not to RTS but rather adapt the level of activity. The primary reason for not returning to sport after ACL revision is the inability to return because of knee-related problems (69%), followed by fear of reinjury (22%) and other reasons (9%) [61–63].

Although the reporting of return to preinjury sport is the most common outcome used, there is an inconsistency in reporting the results on RTS, as the definition used in studies is equivocal [10•, 54, 55, 57, 59, 60, 64, 65]. In a recent systematic review on RTS after ACL revision, the pooled return to any sport rate was 84%, while the return to preinjury sport was 52% and the return to competition was 51% [10•]. In addition, most of the included studies in the review only determined RTS cross sectionally, meaning that patients who were still able to perform their sport make up the return rate, while patients who would have returned but decided to abandon participation were considered as not returned [10•]. The lower proportion of patients returning to their preinjury sport must not imply that treatment after ACL revision is inferior to ACL reconstruction.

#### Objective Measurement Outcomes

With regard to strength and hop testing after primary and revision ACL reconstruction, patients present inferior performance in their injured limb, compared with the healthy limb, regardless of primary or revision ACL reconstruction [66–69]. In a study on 94 patients, ACL revision resulted in greater strength limitations in the injured limb as mean total flexion work (*p* = 0.001), mean total extension work (*p* < 0.001), mean peak flexion torque (*p* = 0.004), and mean peak extension torque (*p* < 0.001) presented deficits, while the primary group only demonstrated limitations in mean peak extension torque (*p* = 0.001) [66]. There are inconclusive results with regard to whether there are differences in peak isokinetic torque of knee extension and knee flexion strength, respectively, between patients who have undergone primary and revision ACL reconstruction [40, 66, 68–70]. However, when comparing percentage loss in muscle strength, patients undergoing ACL revision have been reported to present larger reductions in total flexion work values and total extension work values [66, 68].

In terms of hop testing, the results suggest that there is no difference in recovering symmetry in hop testing between patients undergoing primary and revision ACL reconstruction [67]. Interestingly, there is a greater proportion of patients that do not attempt to perform the hop tests after ACL revision compared with primary ACL reconstruction (on average 24% and 11%, respectively) [67]. In addition, patients undergoing ACL revision required longer time for return to their usual sport compared with patients undergoing primary ACL reconstruction [54]. The clinical implications are to emphasize strength recovery during rehabilitation and help the patients to set proper expectations by informing about the longer time needed to recover after ACL revision. Future research is needed on specific management and adaption in rehabilitation after ACL revision.

## Authors’ Preferred Approach

Our preferred choice in terms of ACL revision is a one-stage procedure, if at all possible. There are several reasons for this, but one-stage procedure is time saving for the patient and orthopedic surgeon as well and the risk of surgical complications is reduced. However, in several cases, a two-stage procedure becomes necessary, especially due to enlarged tunnels in tibia and/or femur which needs to be filled with new bone in order to be able to secure the new graft. Individual choices are therefore often necessary, depending on the size of the tunnels. In many cases, screws or other fixation materials need to be removed, and this may lead to even further enlargement of the bone tunnels and further bone loss, which needs to be addressed.

Allografts are often used; however, allografts are not our first line of treatment in terms of ACL reconstruction. Allografts are weaker than native tissue, and the risk of complications is higher. We mainly use allografts when dealing with multiligament injuries, where the graft material in the patient’s own knee is insufficient, and several ligaments have to be repaired/reconstructed at the same time. A viable option is to use graft material from the contralateral knee. However, many patients are reluctant to harm the healthy knee and prefer allografts instead. Again, individualized decisions are needed.

One important issue is to address all injuries to the knee. This means a thorough evaluation of concomitant meniscus and cartilage injuries and the necessity to take care of additional ligament injuries. In case of rotatory knee laxity, it is insufficient to reconstruct the ACL only. Such a procedure will never be successful and will only lead to inferior PRO and new surgical procedures. All such decisions must be made on an individual level. With regard to graft choice, the QT is a feasible option as it is robust and strong, easily harvested, and a tendon graft of at least 10 mm can be used. This is a major advantage, as the graft size is one decisive factor when it comes to PRO after ACL revision.

Taken together, our preferred choice is QT, and one-stage procedure and all concomitant injuries must be carefully addressed. Malalignment must always be taken care of and has a higher priority than the ligament reconstruction per se.

## Conclusions

Surgical management of revision ACL is complex and constitutes several technical challenges for the orthopedic surgeon. In the pursuit of achieving acceptable results, malpositioned tunnels, tunnel widening, lower limb malalignments, graft choice, and concomitant injuries are factors to be evaluated prior to an ACL revision. With regard to graft choice, allogenous, nonirradiated, and autologous PT, HT, and QT are valid options for ACL revision. Intraarticular chondral pathologies are commonly noted in patients undergoing ACL reconstruction while the presence of concomitant meniscal injuries appears to be lower compared with primary ACL reconstruction. The current evidence demonstrates inferior PRO, lower rates of RTS, and worse functional performance in patients with ACL revision compared with primary ACL reconstruction.
